# Promiscuous evolution of Group A Streptococcal M and M-like proteins

**DOI:** 10.1099/mic.0.001280

**Published:** 2023-01-17

**Authors:** Hannah R. Frost, Julien Guglielmini, Sebastian Duchêne, Jake A. Lacey, Martina Sanderson-Smith, Andrew C. Steer, Mark J. Walker, Anne Botteaux, Mark R. Davies, Pierre R. Smeesters

**Affiliations:** ^1^​ Molecular Bacteriology Laboratory, Université libre de Bruxelles, Brussels, Belgium; ^2^​ Tropical Diseases Research Group, Murdoch Children’s Research Institute, Melbourne, Australia; ^3^​ Institut Pasteur, Université Paris Cité, Bioinformatics and Biostatistics Hub, F-75015 Paris, France; ^4^​ Department of Microbiology and Immunology, University of Melbourne at the Peter Doherty Institute for Infection and Immunity, Melbourne, Australia; ^5^​ Doherty Department, University of Melbourne at the Peter Doherty Institute for Infection and Immunity, Melbourne, Australia; ^6^​ Illawarra Health and Medical Research Institute and School of Chemistry and Molecular Bioscience, University of Wollongong, Wollongong, Australia; ^7^​ Department of Pediatrics, University of Melbourne, Melbourne, Australia; ^8^​ Australian Infectious Diseases Research Centre and School of Chemistry and Molecular Biosciences, University of Queensland, St Lucia, Australia; ^9^​ Academic Children Hospital Queen Fabiola, Université libre de Bruxelles, Brussels, Belgium

**Keywords:** Streptococcus pyogenes, Typing, Virulence, Vaccine, Surface protein, MGA regular

## Abstract

Group A *Streptococcus* (GAS) M and M-like proteins are essential virulence factors and represent the primary epidemiological marker of this pathogen. Protein sequences encoding 1054 M, Mrp and Enn proteins, from 1668 GAS genomes, were analysed by SplitsTree4, partitioning around medoids and co-occurrence. The splits network and groups-based analysis of all M and M-like proteins revealed four large protein groupings, with multiple evolutionary histories as represented by multiple edges for most splits, leading to ‘M-family-groups’ (FG) of protein sequences: FG I, Mrp; FG II, M protein and Protein H; FG III, Enn; and FG IV, M protein. M and Enn proteins formed two groups with nine sub-groups and Mrp proteins formed four groups with ten sub-groups. Discrete co-occurrence of M and M-like proteins were identified suggesting that while dynamic, evolution may be constrained by a combination of functional and virulence attributes. At a granular level, four distinct family-groups of M, Enn and Mrp proteins are observable, with Mrp representing the most genetically distinct of the family-group of proteins. While M and Enn protein families generally group into three distinct family-groups, horizontal and vertical gene flow between distinct GAS strains is ongoing.

## Data summary

The authors confirm all supporting data, code and protocols have been provided within the article or through supplementary data files. The multiple sequence alignment files and correspondence between designated protein alleles and DNA sequences are publicly available on the figshare website (https://figshare.com/s/6c488562c319f800d13d).

## Introduction

As a human-specific pathogenic bacteria, the Group A *Streptococcus* (GAS; *Streptococcus pyogenes *) has evolved a diverse array of surface proteins and virulence factors that enable colonisation and immune evasion [1]. Although GAS is one of the leading causes of death from infectious diseases globally [2], the vast strain diversity and global epidemiological differences have hindered development of an effective vaccine [3, 4].

The principle measure employed to manage the species diversity, is the use of typing systems to classify the bacteria into strains. Typing systems for GAS include: M or *emm-*typing, based on the hypervariable region (HVR) of the M protein [5, 6], *emm*-pattern typing based on composition of *emm* and *emm*-like genes [7, 8], *emm*-cluster typing based on evolutionary and functional properties of M proteins [9], T typing based on pilus proteins [10, 11], multi-locus sequence typing (MLST) based on sequences of seven house-keeping loci [12] and whole genome clusters based on core genome content [13].

Typing strains based on the M protein has functional relevance as the M protein is an essential virulence factor of GAS and M proteins are target antigens of many leading vaccine candidates [14]. However, the characterisation of over 240 *emm-*types and over 1000 *emm* subtypes complicates studies of functional and immunological properties of M proteins and development of broadly efficacious vaccines. Grouping *emm*-types into functionally relevant *emm*-clusters provides a framework for immunological analyses of M proteins, as demonstrated by evidence of cross-reactive immunity within clusters [15].

Immediately upstream and downstream of the *emm*-gene in 85 % of GAS isolates are the genes encoding the M-like proteins Mrp and Enn [16]. These proteins are similar to M proteins in both structure and function; all are surface expressed coiled-coiled dimers with shared binding capacities for host proteins [17]. Another M-like protein, Protein H, is encoded by the *sph* gene downstream of the *emm* gene in a restricted number of *emm*-types [16]. Protein H shares functionality with both M and Enn proteins [18, 19]. Evidence in *emm*4 [20] and several other *emm*-types [16] indicates that *emm* and *enn* genes may recombine by homologous recombination to form chimeric M/Enn proteins. These data suggest that the evolutionary histories of *emm*-like genes may be fluid, a product of both their close genetic context and similar structural and functional properties. Homologous recombination is a common way by which GAS maintains genetic diversity [13] and this has been observed to occur between GAS strains [21] and between streptococcal species [22].

In this study, we investigated the genetic relationships within the M and M-like protein families and provide an improved framework to elucidate the functional and biological associations of these dynamically evolving proteins.

## Methods

### Database and phylogenetic inferences of M and M-like family of proteins

**Fig. 1.**
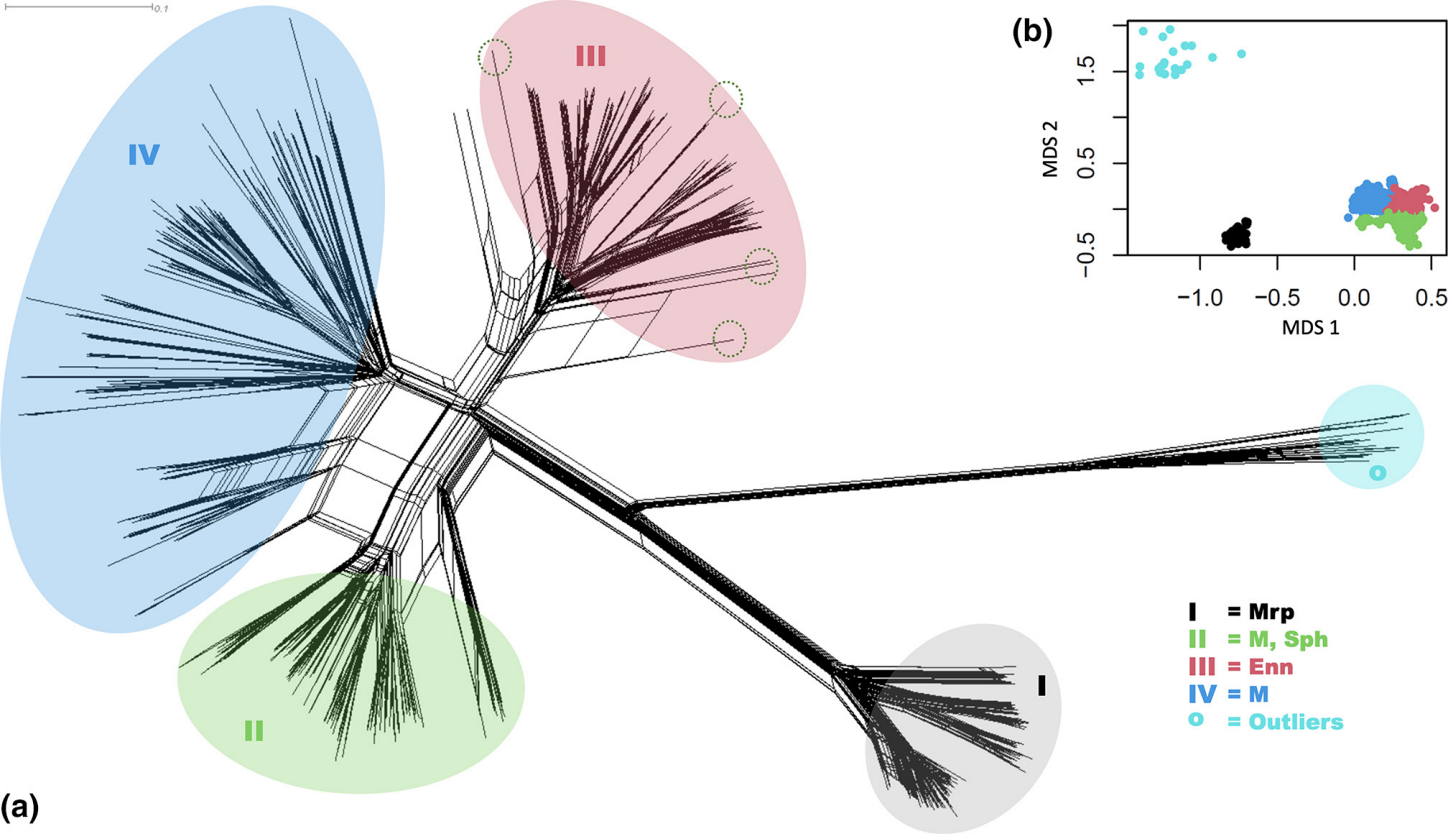
SplitsTree network and partitioning around medoids grouping of M and M-like proteins. (**a**) SplitsTree4 (version 4.15.1) was used to generate a neighbour-net genetic network based on the alignment of 1054 M and M-like protein sequences using the uncorrected p-distance. The coloured ellipses indicate the assigned M-family-groups of the sequences as defined in (**b**), with relative position of chimeric M proteins indicated by dotted lines. (**b**) M-family-group predictions were assigned using the partitioning around medoids algorithm, and four M-family-groups plus a group of outlier proteins were identified based on genetic distance by multidimensional scaling (MDS). The two nodes not included in either ellipse represent a Protein H allele (left) which belongs to family group IV, and an emm137 protein (right) which belongs to family group 3.

The *emm*, and *emm*-like (*enn, mrp* and *sph*) genes from a genetically diverse collection of 1668 contiguous Mga regulons representing 130 different *emm*-types and 39 *emm*-clusters were extracted based on genetic probes, open reading frame predictions and sequence similarity to published gene sequences [13, 16]. Sequences of *emm* and *emm*-like genes were translated and the signal peptide predicted using the SignalP-5.0 server [23]. Mature sequences were generated by *in silico* removal of the signal peptide up to the cleavage site and from the glycine residue of the LPXTG-sortase motif, used to attach the protein to the bacterial surface. The final database of M and M-like protein sequences analysed in this study contains 1054 unique proteins, comprising of 541 M, 228 Mrp, 275 Enn and ten Protein H sequences. Multiple sequence alignments (MSA) of unique protein sequences were generated using MAFFT version 7.311 using the G-INS-i method which performs global alignments using the Needleman-Wunsch algorithm [24]. The MSA of 1054 M and M-like proteins identified 1552 total sites (ungapped length mean=321.4, Std Dev=48.2) including 30 complete sites, 20 variable sites and 16 informative sites. The M protein MSA had 1404 total sites (ungapped length mean=336.0; Std Dev=58.1) including 60 variable sites and 51 informative sites. The Mrp protein MSA had 377 total sites (ungapped length mean=326.2; Std Dev=19.5) including 222 complete sites, 133 variable sites and 107 informative sites. The Enn protein MSA had 487 sites (ungapped length mean=287.9; Std Dev=17.0) including 92 complete sites, 48 variable sites and 40 informative sites. As M and M-like proteins display recombinogenic potential, networks were inferred using SplitsTree4 (version 4.15.1) using uncorrected p-distance and the neighbour-net analysis [25] and consequently do not incorporate evolutionary models. The SplitsTree networks were used to define ‘groups’ of M, Mrp or Enn proteins alone and overlaid with ‘M-family-groups’ and ‘sub-groups’. Outliers from each family were observed in the all protein SplitsTree network and divergence in MSA and removed (seven Mrp, four M and 13 Enn proteins; Fig. 1, Table S1).

### Protein group prediction

Partitioning around medoids algorithm, implemented in RStudio (version 3.6), was utilised to estimate the assignment of M and M-like proteins to ‘M-family-groups’ and the assignment of M, Mrp and Enn proteins to ‘sub-groups’. Partitioning around medoids is based on the p-distance between samples and has the benefit of not assuming a tree-like structure linking all the data points, yet is limited in that it does not reflect ancestry, indicating that assigned groups are not fixed and should be interpreted as groupings based on statistical fit. To choose the optimal number of groups we used partitioning around medoids for *k*=1 to *k*=*n*-1, where *k* is the possible number of groups and *n* is the number of data points, and selected the *k* value with the best GAP score. Importantly, this M-family-group and M, Mrp and Enn sub-group approach requires a matrix of coordinates instead of actual distances, for which we used the multidimensional scaling of the evolutionary distances of the MSA above. MSA of groups and sub-groups were performed as above and groups features plotted with GraphPad Prism version 7.0.

### Co-occurrence network

For co-occurrence analyses, genomes that did not contain an *mrp* or *enn* were removed from *enn* and *mrp* network analyses respectively. Where more than one isolate of an *emm*-type was available (*n*=110) the different combinations of alleles were used to generate co-occurrence networks using the igraph software package [26] with RStudio [27]. The node sizes are proportionate to number of occurrences of each allele and the edge weight is proportionate to the number of occurrences of each unique combination of alleles. Community detection was performed using the label propagation algorithm, a clustering method which maximises the internal density of communities [28].

## Results

We define multiple levels of protein grouping herein, and to avoid confusion and redundancy with previous publications, we use the terms: ‘M-family-groups’ when referring to divisions between the different M and M-like proteins altogether, ‘groups’ when referring to specific SplitsTree network groupings of M, Mrp or Enn proteins alone, and ‘sub-groups’ when referring to partitioning around medoids-defined groupings of M, Mrp and Enn proteins alone.

### M and M-like proteins form four distinct M-family-groups with high evolutionary plasticity

In a recent analysis of global GAS genomes, we defined a database of 1688 Mga regulons [13, 16]. In order to define the genetic relationship of M and M-like proteins within this database, we defined 1054 unique protein sequences comprising of 537 M-proteins and 493 M-like proteins (221 Mrp, 262 Enn, and 10 Protein H), and 24 outlier proteins. The splits network of all 1054 M and M-like proteins revealed several large protein groupings with multiple evolutionary histories as represented by multiple edges for most splits, leading to four ‘M-family-groups’ of protein sequences with a fifth grouping containing 24 outlier proteins (Fig. 1 and Table S1). Such net-like splits structure is indicative of evolutionary incongruence, possibly driven by recombination. Mrp proteins formed a single clearly defined ‘M-family-group I’ relative to the other M-like genes. M-family-group III included Enn proteins and previously reported chimeric Enn-M proteins. M-family-group IV contained only M proteins while M-family-group II included both M protein and Protein H, a rare variant of M-like proteins. When overlaid with *emm*-clusters [9], M-family-group IV contained all clade Y and E6 cluster M proteins and M-family-group II contained E1, E2, E3 and E4 M proteins [9]. These data indicate that Mrp proteins are more genetically distant from the other M and M-like proteins, with recombination more likely to occur within the Mrp M-family group than between Mrp and other M and M-like proteins. By contrast, M and Enn proteins appear closely related to each other with shorter genetic distances and higher potential for recombination (Fig. 1). To further examine the phylogenetic network of this dynamic family of M- and M-like proteins, we built splits networks for each of the three major protein families (M, Mrp and Enn).

### M proteins form two distinct groups, containing nine sub-groups

**Fig. 2.**
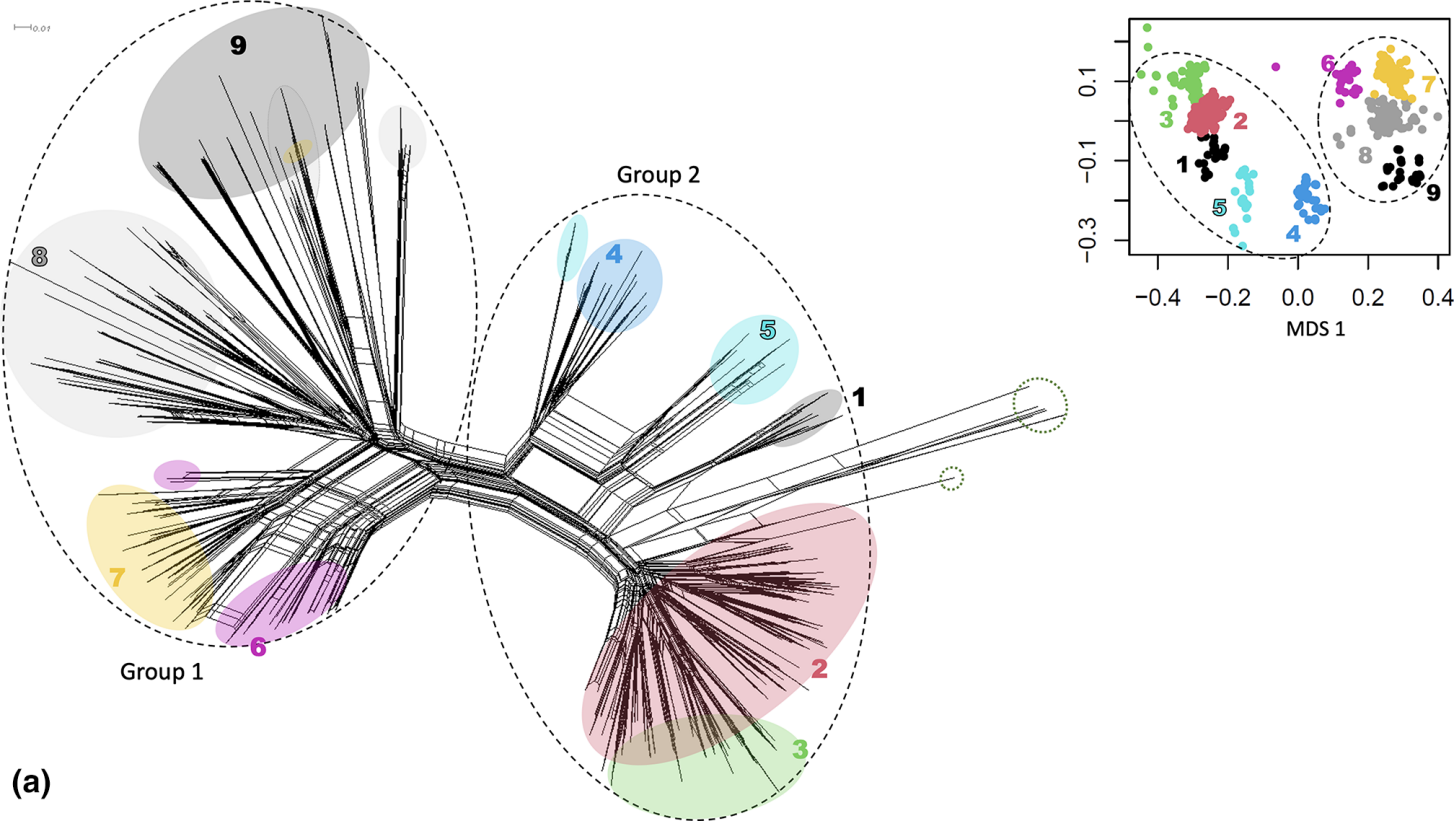
SplitsTree network and partitioning around medoids grouping of M proteins. (**a**) SplitsTree4 (version 4.15.1) was used to generate a neighbour-net genetic network based on the alignment of 537 M protein sequences using the uncorrected p-distance. The black dotted ellipses indicate the two M protein groups, green dotted ellipse indicates chimeric M proteins, and coloured ellipses indicate the assigned M sub-groups, as defined in (**b**). (**b**) Group predictions were assigned using the partitioning around medoids algorithm, and nine M sub-groups were identified based on genetic distance by multidimensional scaling (MDS) and overlaid on the SplitsTree network.

The SplitsTree network of 537 M proteins revealed a complex evolutionary relationship where parallel edges define groups of related proteins (Fig. 2). Group analysis revealed two major ‘groups’ which contained nine ‘sub-groups’ (Fig. 2, Fig S1, Table S2). These groupings are largely congruent with the previously published functional designations of clades X (group 2) and Y (group 1) (Fig. S2) [9], with some deviations. Group 1 M proteins contain four subgroups (subgroups 6–9) which have more overlap than the five subgroups contained in Group 2. Subgroups 4 and 5 appear more separate from the remaining Group 2 subgroups, and comprise all E6 emm cluster M proteins. It is interesting to note that the E6 *emm* cluster contains C-terminal sequence consistent with Clade Y proteins, but was phylogenetically and functionally closer to the remainder of clade X proteins. As previously described, *emm* cluster E6 appears an intermediary between the two clades [9]. M proteins have previously been differentiated into two distinct *emm* subfamilies by Hollingshead *et al*., based largely on signal peptide sequences. Signal peptide sequences were not included in our MSA, however the grouping predictions retain the two distinct groups [8, 29].

### Mrp proteins form four specific Mrp groups with ten Mrp sub-groups

**Fig. 3.**
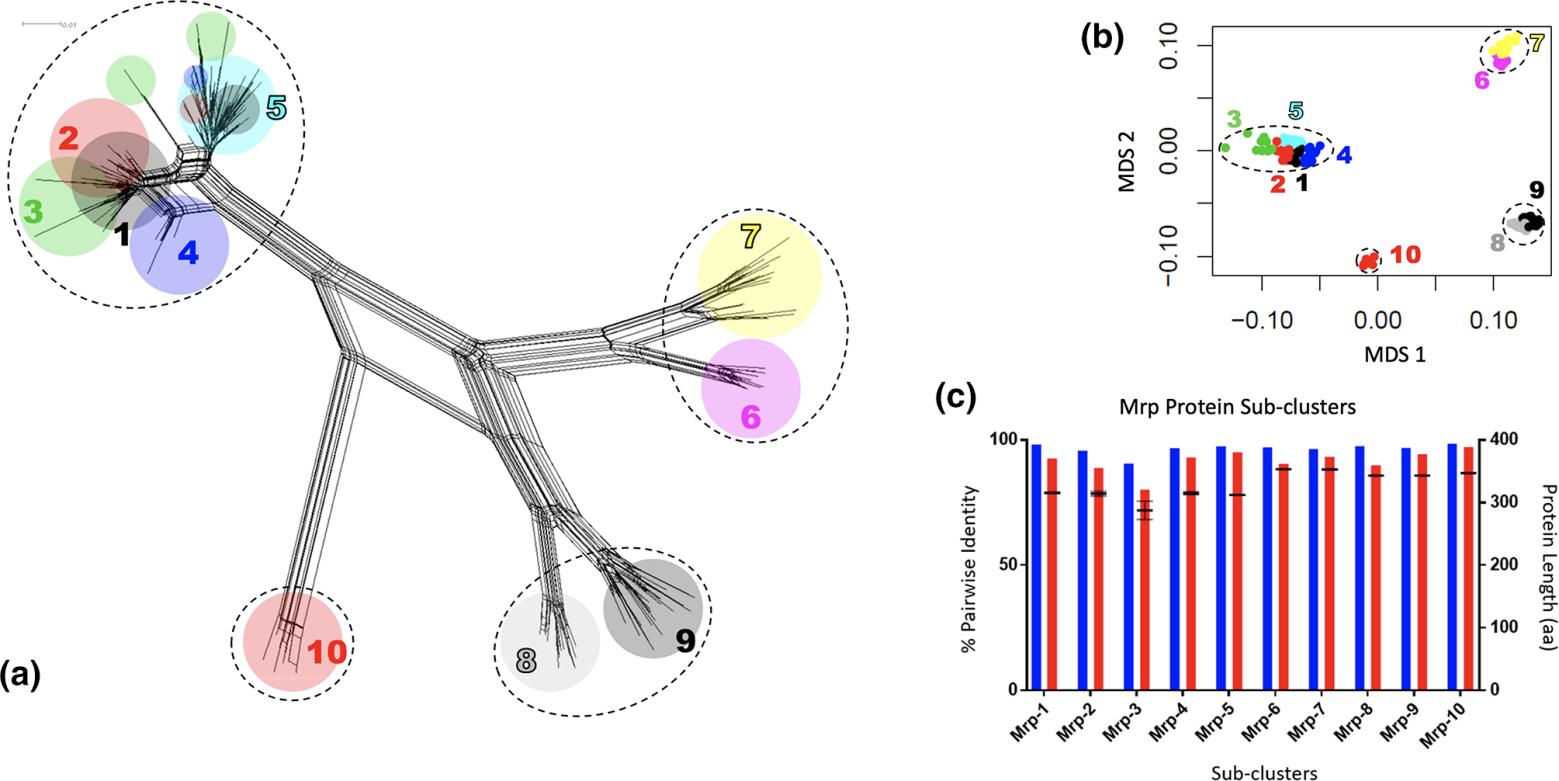
Sub-grouping of Mrp proteins. (**a**) Neighbour-net genetic network based on 221 Mrp protein sequences using the uncorrected p-distance in SplitsTree4 (version 4.15.1). Dashed lines represent four Mrp specific groups. The coloured ellipses refer to the ten assigned sub-groups of Mrp sequences based on genetic distance as defined by multidimensional scaling (MDS) (**b**). Sub-groups do not always reflect evolutionary independent pathways (example 1–5) reflecting a complex and evolving evolution history. (**c**) MDS Mrp sub-groups have very high average (blue bars) and minimum (red bars) sequence identity, and the mean protein lengths are highly similar (black symbols indicating mean protein length with range).

Analyses of Mrp proteins revealed the presence of four specific groups (Fig. 3) with evidence of a further ten sub-groups based on multidimensional scaling and partitioning around medoids grouping (Fig. 3, Table S3). Around half of the proteins (130/221) form groups of multiple Mrp sub-groups (SG) that are not congruent with network tree topology (Fig. 3; Mrp sub-group 1–5) whereas the other proteins (90/221) coincide with more structured branching patterns in the network, influenced by multiple edges in the splits network (Mrp sub-group 6–10). These differences may be attributed to the different weightings afforded to different variable or constant sites within MSAs between the SplitsTree and partitioning around medoids algorithms. The average and minimum sequence identity within each sub-groups (Table 1, Fig. 2c) is high, and aside from group Mrp sub-group 3, the protein lengths within a group are similar.

**Table 1.** Details of Mrp and Enn sub-groups

	#unique proteins	% average pairwise identity	%minimum identity	Mean protein length (aa)	Length std dev
Mrp-SG1	41	97.9	92.4	315.4	1.5
Mrp-SG2	27	95.7	88.6	314.0	3.8
Mrp-SG3	13	90.5	80.1	287.2	14.4
Mrp-SG4	15	96.6	93.0	314.7	2.4
Mrp-SG5	34	97.4	94.9	312.0	0.2
Mrp-SG6	16	96.9	90.4	353.0	0.0
Mrp-SG7	23	96.2	93.2	352.6	0.5
Mrp-SG8	19	97.5	89.8	343.0	0.0
Mrp-SG9	25	96.8	94.2	343.0	0.0
Mrp-SG10	8	98.4	97.1	347.0	0.0
Enn-SG1	40	90.0	72.8	298.7	13.9
Enn-SG2	17	92.4	74.6	296.7	14.0
Enn-SG3	40	92.6	78.8	293.8	9.7
Enn-SG4	13	88.2	67.5	250.2	9.2
Enn-SG5	46	87.0	64.4	285.2	9.2
Enn-SG6	23	92.7	75.0	262.1	10.4
Enn-SG7	30	81.4	63.7	292.0	12.8
Enn-SG8	25	82.1	69.2	296.9	8.3
Enn-SG9	28	81.0	67.2	289.3	10.3

### Enn proteins form two Enn groups with nine Enn sub-groups

**Fig. 4.**
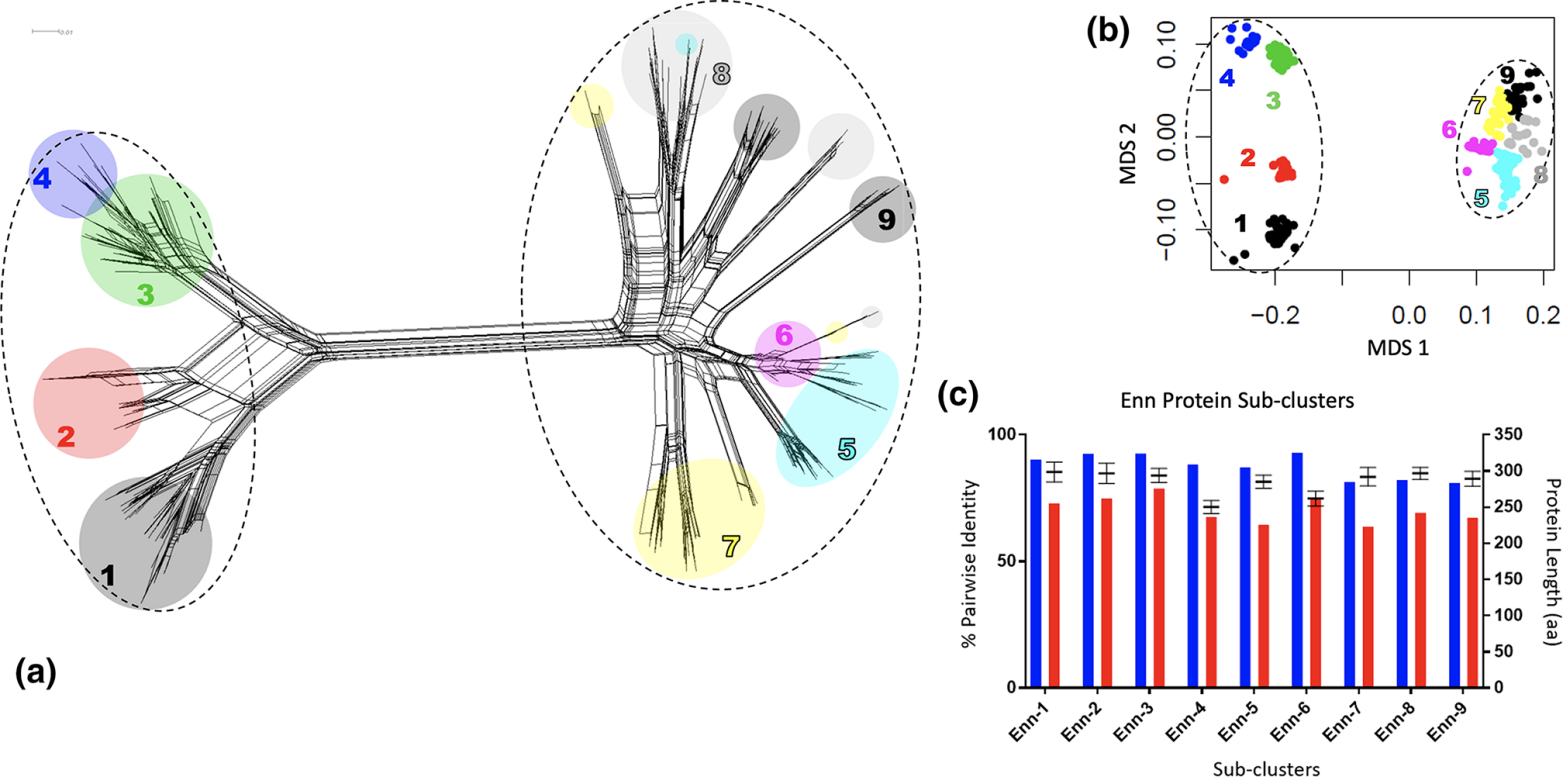
Sub-grouping of Enn proteins. (**a**) Neighbour-net genetic network based on 262 Enn protein sequences using the uncorrected p-distance in SplitsTree4 (version 4.15.1). Dashed lines represent two Enn specific groups. The coloured ellipses refer to the nine assigned sub-groups of Enn sequences based on genetic distance as defined by multidimensional scaling (MDS) (**b**). Similar to other M-like proteins (eg. Mrp, Fig. 2), Enn sub-groups reflect a complex and distorted evolution history. (**c**) There is high average sequence identity (blue bars) within MDS Enn sub-groups, although the minimum identity (red bars) is lower than observed with Mrp, potentially due to the greater variability in protein lengths observed (black symbols indicate mean protein length with range).

Similarly to M proteins, the genetic network of Enn proteins reveals two groups, with evidence of up to nine sub-groups based on multidimensional scaling that exhibit convoluted evolutionary histories (Fig. 4;Table S4). All sub-groups are represented by multiple Enn proteins with multiple splits networks between nodes of different sub-groups. The average identity within Enn sub-groups is high, although the minimum identity is lower than observed for Mrp sub-groups (Table 1, Fig. 3c), likely due to the greater variability observed within Enn proteins [16]. The variability between protein lengths within a sub-group is relatively low.

### Recombination between *emm* and *enn* has resulted in new gene families

**Fig. 5.**
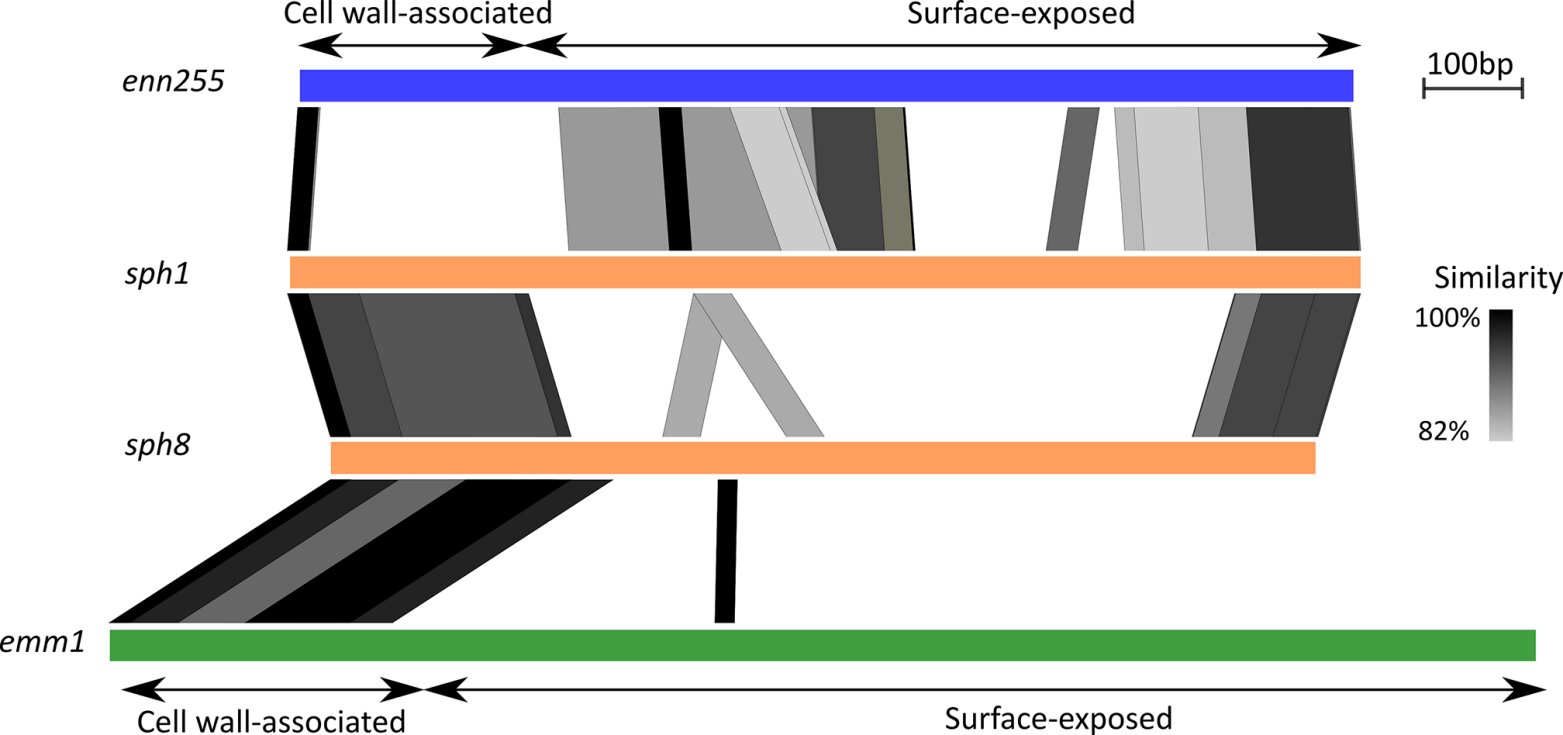
Emergence of *sph* gene family through *emm* and *enn* recombination. Pairwise alignment of representative *enn*, *sph* and *emm* genes showing that the *sph* gene product likely arose from ancestral recombination between *emm* and *enn* genes. *enn255* (blue) and *sph1/sph8* (orange) genes share higher sequence similarity across the surface-exposed N-terminal domain, while the C-terminal cell wall-associated regions are syntenic between *sph1/sph8* and Clade Y *emm* (represented here as *emm1* - green) genes. *sph* genes exhibit high levels of sequence variation across central domain regions. Pairwise alignment was generated using Easyfig with Blast similarity (tblastx) denoted by the gradient bar.

Splits network suggests that recombination has played a role in the evolution of the M-like family of proteins, in particular between *emm* and *enn* groups where intermediate splits resulting in distinct gene groups are observed. However without a historical, longitudinal analysis of related strains it is difficult to determine the extent of recombination and other episodes of horizontal gene transfer in a context of host immune diversifying pressure on those surface exposed proteins. As an exemplar of the complex evolution history between M-like protein families, alignment of major sub-groups identified evidence of recombination events between *emm* and *enn* resulting in the formation of the gene family corresponding to *sph*, encoding Protein H. The overall structure of all ten *sph* alleles analysed in this study were similar even though high sequence variation existed within central domains of *sph* genes (Fig. 5). Alignment of two allelic forms of *sph* revealed high synteny (>98 %) with ~300 nucleotides of the cell wall associated C-terminal of *emm*1 (Clade Y), while the N-terminal of all *sph* in this dataset share >92 % synteny over ~120 nucleotides with the surface exposed N-terminal of *enn205* (Fig. 5). The central portion of *sph* is genetically variable, as is common across M-family proteins. The known host protein interactions attributed to Protein H occur within the C-repeat and N-terminal regions [30, 31] meaning these functions most likely derive from the Enn precursor. These data indicate that Protein H is likely a chimeric descendent of an ancestral recombination event between the *emm* and *enn* gene families followed by diversification within the central repeat regions, leading to a new chimeric *emm*-like gene family that has been maintained within a subset of the GAS population. Other recombination events between *emm* and *enn* genes in this collection has generated chimeric *emm* genes from multiple different lineages, as has been reported previously [16, 20].

### *mrp* and *enn* alleles are co-inherited with *emm* alleles

**Fig. 6.**
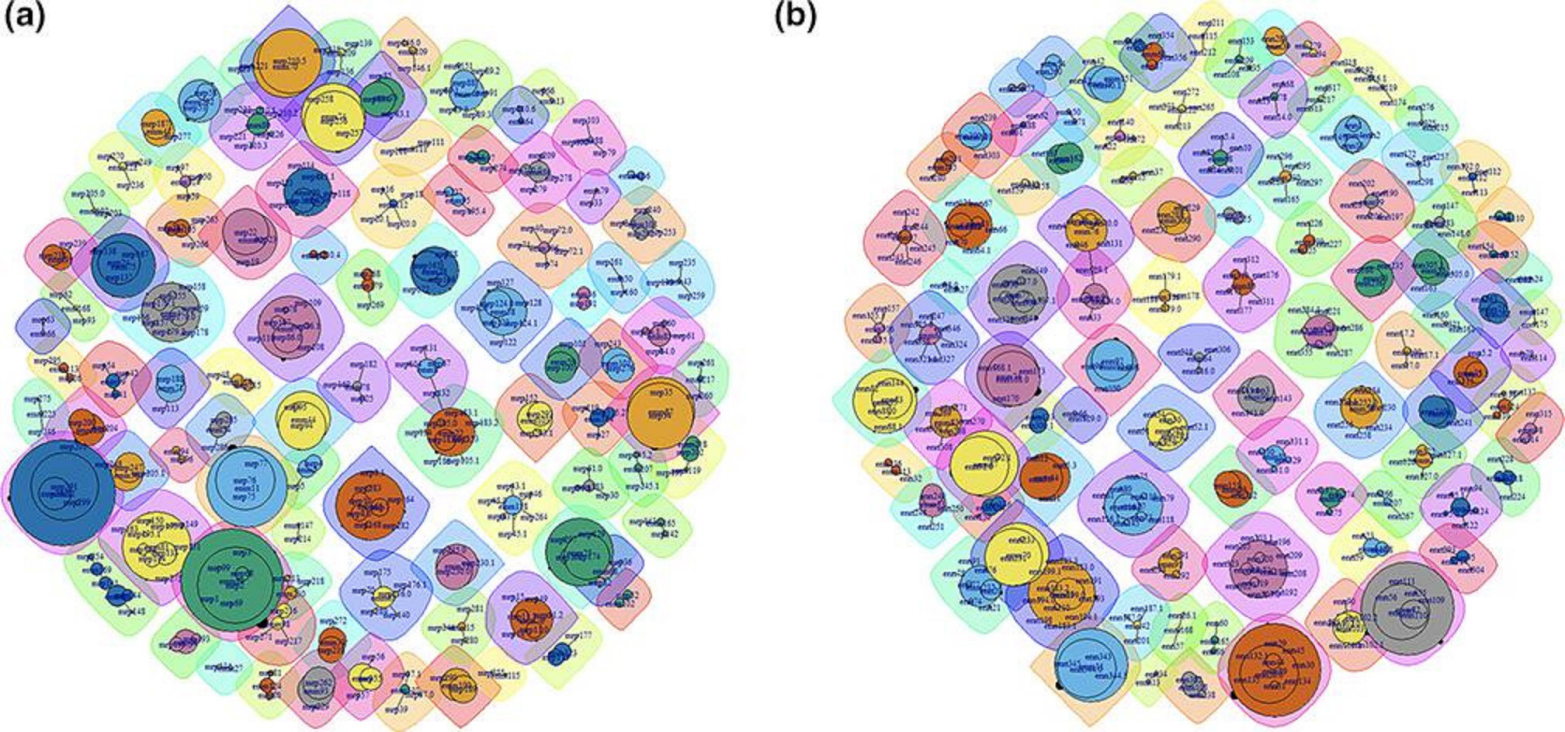
Co-occurrence networks of *emm*-types with *mrp* or *enn allelles*. Co-occurrence networks of *emm* types with *mrp* (**a**) or *enn* (**b**) alleles, generated with igraph. The largest association in both networks is comprised of those *emm*-types with no *mrp* or *enn* allele (*emm* only). Communities were detected with label propagation and had high modularity (emm +mrp=0.9619, emm +enn=0.9718). These networks and communities show that there are discrete associations between a limited number of *mrp* or *enn* alleles with each *emm*-type, with up to 109 and 110 communities respectively. Communities are distinguished by both node and surrounding cloud colour.

Analysis of the co-occurrence of the different *mrp* and *enn* alleles with each *emm*-type in the collection revealed discrete groupings of small numbers of different alleles, rather than many connections between many alleles (Fig. 6). In the collection of unique genes, there were 130 *emm*-types, 110 of which were represented by more than one isolate. Community detection found 109 *emm +mrp* and 110 *emm +enn* communities, much lower than the possible combinations between *mrp* or *enn* alleles and *emm*-types, which is evidence against unrestricted recombination driving the evolution of the M-like family. The edge density (proportion of combinations of alleles from all possible combinations) was 0.004 for both *emm +mrp* and *emm +enn*. Between *mrp* and *enn* alleles from those genomes that contained both, there were 198 communities detected (modularity=0.98; Fig. S3a). These networks suggest the inheritance of alleles is restricted around *emm*-types, indicating high general concordance between the co-inheritance of *emm* and *emm*-like genes. Of note, the variability between genes within communities varied, with some *emm*-types linked with >5 *mrp* or *enn* genes of the same sub-group (e.g. *emm*58, *emm*81), and other *emm*-types linked with *emm*-like genes from >3 sub-groups (e.g. *emm*60, *emm*114, *emm*122). As the *emm*-cluster system was defined based on different phylogenetic algorithms to those used herein and functional properties of proteins [9], the proposed *mrp* and *enn* specific groups and sub-groups do not directly compare. However, broadly there appeared to be more homogeneity within co-inherited *enn* sub-groups than *mrp* sub-groups within each *emm*-cluster. The associations of *mrp* and *enn* sub-groups with *emm*-clusters was further analysed by co-occurrence network, which revealed similar associations with more moderate statistical support (modularity 0.41 and 0.43 respectively; Fig. S3b, c).

## Discussion

The Mga regulon, essential for virulence and epidemiology of GAS, contains the highly variable yet functionally related *emm* and *emm*-like gene families, representing a complex locus for genetic investigation [16]. We aimed to resolve the observed high levels of sequence diversity to that which may be biologically relevant, establishing a manageable framework for further investigation, by grouping related M and M-like protein sequences. Our findings highlight the complex and dynamic framework of *emm* and *emm*-like gene families. At a granular level, distinct networks of M, Enn and Mrp proteins are observable with Mrp representing the most genetically distant of the family of proteins. While M and Enn protein families generally group into three distinct populations, extensive horizontal and vertical gene flow is possible, through both homologous recombination events, transduction and selection pressures combined with a high level of mutation.

The extent of recombination within and between the different families of *emm* and *emm*-like genes remains unknown, and would require longitudinal rather than cross-sectional analysis. However, there were identifiable recombination events leading to the establishment of known chimeric M proteins (M4, M9, M44, M58, M73, M82) [16] and Protein H within the global population of GAS bacteria. Interestingly, the chimeric M proteins grouped within the Enn protein division (M-family-group 3) in the SplitsTree network, and Protein H appear as a sub-population between the Enn and M protein divisions (M-family-group II and III). Protein H is found in the majority of M1 isolates in certain geographical populations [18] however in this collection, Protein H was restricted to emm19.4, emm238 and emm57 isolates [16] which represents a limitation of the data used in this study. Chimeric M proteins were present less frequently, though in multiple lineages. Therefore, the epidemiological impact of these successful recombination events remain unclear.

The mosaic evolutionary structure observed within M and M-like protein families may also be driven by selective pressures and convergent evolution that select for functional attributes. There is support for the existence of sub-populations of Mrp and Enn proteins on the basis of distance-based grouping approaches as applied in this study; however, these group designations are not iterative, with groupings dependent on database and distance matrices. Whether these sub-groups relate to functionally or biologically constrained populations requires future investigations, yet this approach provides a robust framework to facilitate targeted studies. Such sub-grouping does not reflect evolutionary trajectory given the extensive plasticity within phylogenetic networks, yet reflects adaptive or convergent evolution over a time.

Within this evolutionary framework, we reveal modules of co-inherited alleles, with limited combinations of *mrp* and *enn* alleles occurring with each *emm*-type. This suggests an overarching stability within the Mga locus as a unit, despite ongoing flow of genetic material. In this global collection of GAS genomes, we determined 93 combinations of *mrp* and *emm*-type, and 95 combinations of *enn* and *emm*-type. The co-inheritance of specific alleles has functional implications, as different variants of Mrp and Enn proteins are known to perform different functions [17], as are different M proteins [14]. It is therefore possible that the restriction to the observed co-inherited proteins is due to a combinatorial effect on virulence, as is observed with other groups of virulence factors [32]. Apparent associations between *mrp* and *enn* alleles with specific *emm*-clusters provide attractive preliminary evidence of a functional basis for co-inheritance. However as the M family grouping systems proposed herein and the established *emm*-cluster system derive from different data, algorithms, and has only been validated experimentally for the *emm*-clusters, any substantial association requires experimental validation for the M-like (sub)groups as well.

The evolution of this genetically and functionally related family of gene products is ongoing, and this study provides a snapshot of this dynamic process. Gene flow, both large and small scale, has the potential to change the functionality of these virulence factors and the virulence potential of the bacteria. An improved understanding of GAS population genetics will provide a better understanding of GAS biology, particularly important in the context of vaccine development. With antigenic drift a risk factor for vaccine efficacy, the close relationship between M and M-like proteins should be considered in the development of any M protein-based vaccine. These groups and sub-groups provide a framework for functional characterisation which will determine whether these divisions are functionally distinct.

## Supplementary Data

Supplementary material 1
