# Supplementary material for: Promiscuous evolution of Group A Streptococcal M and M-like proteins
Source: Microbiology (Reading). 2023 Jan 17;169(1):001280. doi: 10.1099/mic.0.001280 (PMC9993116; doi:10.1099/mic.0.001280)

Supplementary material

Promiscuous evolution of M and M-like proteins

Hannah R. Frost[a,b], Julien Guglielmini[c], Sebastian Duchêne[d], Jake Lacey[d], Martina Sanderson-Smith[e], Andrew C. Steer[b,f], Mark J. Walker[g], Anne Botteaux[a], Mark R. Davies[d]*and Pierre R. Smeesters[a,b,f,h]*

*Contributed equally

Supplementary Table 1: Details of Outlier proteins

**Mrp Outliers:**		**Emm Outliers:**		**Enn Outliers:**	
mrp1	77aa insertion	emm39.4_266	Frameshift mutation	enn283	C-terminal sequence discrepancies
mrp15	35aa deletion	emm90.5_525	Frameshift mutation	enn282	C-terminal sequence discrepancies
mrp110	35aa deletion	emm110.0_615	Frameshift mutation	enn350	C-terminal sequence discrepancies
mrp111	N-terminal deletion	emm75.1_416	C-terminal sequence discrepancies	enn353	C-terminal sequence discrepancies
mrp113	N-terminal deletion			enn280	C-terminal sequence discrepancies
mrp114	N-terminal deletion			enn281	C-terminal sequence discrepancies
mrp186	35aa deletion			enn159	Doesn't align
				enn158	Doesn't align
				enn160	Doesn't align
				enn161	Doesn't align
				enn151	C-terminus truncated
				enn349	C-terminal sequence discrepancies
				enn228	Doesn't align

Table 1: list of the outlier proteins removed from each protein family before phylogenetic analysis and brief reasoning for removal. The C-terminal sequence discrepancies were manually identified in the MSA.

Supplementary Table 2: PAM-derived sub-groups of *emm* genes

emm sub-group	*emm* alleles
SG1	emm60, emm169, emm78, emm176, emm165, emm4, emm77
SG2	emm60, emm92, emm76, emm13, emm117, emm110, emm106, emm27, emm44, emm113, emm90, emm104, emm68, emm168, emm102, emm73, emm114, emm124, emm2, emm77, emm112, emm232, emm84, emm126, emm89, emm109, emm22, emm28, emm8, emm88, emm50, emm183, emm9, emm49, emm151, emm118, emm15
SG3	emm76, emm44, emm113, emm25, emm103, emm87, emm79, emm209, emm58, emm82, emm66, emm90, emm168, emm9, emm73
SG4	emm59, emm65, emm99, emm85, emm42, emm81, emm185, emm191
SG5	emm63, emm94, emm11, emm177, emm75, emm48
SG6	emm137, emm230, emm225, emm70, emm93, emm224, emm192, emm64, emm56, emm223, emm43, emm83, emm119, emm116
SG7	emm230, emm225, emm108, emm33, emm86, emm93, emm41, emm83, emm52, emm98, emm80, emm101, emm43, emm91, emm178, emm53, emm162, emm186, emm119, emm6, emm97, emm184, emm147
SG8	emm41, emm18, emm207, emm54, emm179, emm218, emm19, emm6, emm36, emm71, emm100, emm115, emm32, emm197, emm217, emm123, emm5, emm105, emm74, emm14, emm233, emm122, emm97, emm57, emm1, emm227, emm238, emm29
SG9	emm24, emmstG3251, emm111, emm221, emm57, emm3, emm12, emm229, emm39, emm55, emmstg1750, emm222, emm95

Table 2: emm sub-group allele lists. Full protein sequences were used to define clusters. Unlike mrp and enn designations, *emm* typing is based on a small portion of the full sequence, therefore allowing sequence diversity within a type, which is reflected in the same *emm* types present in >1 sub-group.

Supplementary Table 3: PAM-derived sub-groups of *mrp* genes

Mrp sub-group	Mrp alleles
SG1	mrp193.0, mrp193.1, mrp193.2, mrp193.3, mrp193.4, mrp193.5, mrp193.6, mrp193.7, mrp193.8, mrp193.9, mrp193.10, mrp193.11, mrp195.0, mrp195.1, mrp195.2, mrp195.3, mrp195.4, mrp195.5, mrp195.6, mrp195.7, mrp195.8, mrp195.9, mrp199, mrp201, mrp202, mrp203, mrp205.0, mrp205.1, mrp205.2, mrp209, mrp211, mrp213, mrp214, mrp221, mrp223, mrp226, mrp232, mrp235, mrp236, mrp237, mrp240, mrp241, mrp242, mrp243, mrp244, mrp245.0, mrp245.1, mrp245.2, mrp249, mrp254, mrp255, mrp256.0, mrp256.1, mrp260, mrp261, mrp264, mrp268.0, mrp268.1, mrp270, mrp275
SG2	mrp216.0, mrp216.1, mrp217, mrp219, mrp229, mrp230.0, mrp230.1, mrp246, mrp247, mrp248, mrp257, mrp258, mrp262, mrp265, mrp267.0, mrp267.1, mrp278, mrp279, mrp280, mrp281, mrp282.0, mrp282.1, mrp285, mrp286, mrp287, mrp288, mrp289, mrp152, mrp158, mrp161, mrp164
SG3	mrp253, mrp271, mrp272, mrp273, mrp274, mrp284, mrp290, mrp121, mrp128, mrp138, mrp160, mrp165, mrp166
SG4	mrp178, mrp179.0, mrp179.1, mrp179.2, mrp182, mrp187, mrp188, mrp189, mrp190, mrp191, mrp192, mrp171, mrp172, mrp173, mrp174, mrp176.0, mrp176.1, mrp176.2, mrp177
SG5	mrp115.0, mrp115.1, mrp115.2, mrp115.3, mrp117, mrp118, mrp119, mrp122, mrp123, mrp124.0, mrp124.1, mrp126, mrp127, mrp129, mrp131, mrp132, mrp133, mrp136.0, mrp136.1, mrp137, mrp139, mrp140, mrp141, mrp144, mrp145, mrp146.0, mrp146.1, mrp149, mrp150, mrp151, mrp153, mrp154, mrp155.0, mrp155.1, mrp156, mrp159, mrp162, mrp163, mrp167, mrp169, mrp170
SG6	mrp2, mrp3, mrp5.0, mrp5.1, mrp6, mrp7, mrp8.0, mrp8.1, mrp8.3, mrp11.0, mrp11.1, mrp11.2, mrp14, mrp16, mrp17, mrp19, mrp20.0, mrp20.1, mrp22, mrp23, mrp25, mrp28
SG7	mrp24, mrp26, mrp27, mrp29, mrp30, mrp31.0, mrp31.1, mrp33, mrp34.0, mrp34.1, mrp36, mrp37.0, mrp37.1, mrp39, mrp40, mrp41, mrp42, mrp43.0, mrp43.1, mrp45.0, mrp45.1, mrp46, mrp48, mrp49, mrp50, mrp52, mrp53, mrp54
SG8	mrp88, mrp89.0, mrp89.1, mrp89.2, mrp89.3, mrp91, mrp93, mrp94.0, mrp94.1, mrp96, mrp97, mrp98, mrp99, mrp100, mrp101, mrp102, mrp103, mrp104, mrp105.0, mrp105.1, mrp107, mrp108, mrp109, mrp83.0, mrp83.1
SG9	mrp55, mrp56, mrp57, mrp58, mrp59, mrp61.0, mrp61.1, mrp62, mrp63, mrp64.0, mrp64.1, mrp66, mrp67, mrp68, mrp69, mrp70, mrp71, mrp72.0, mrp72.1, mrp72.3, mrp75, mrp76, mrp77, mrp78, mrp79, mrp80.0, mrp80.1, mrp82, mrp85, mrp86.0, mrp86.1
SG10	mrp291, mrp292, mrp293.0, mrp293.1, mrp295, mrp296, mrp297, mrp298, mrp299

Table 3: mrp sub-group allele lists. Protein sequences were used to define clusters, but alleles which have the same mature protein sequences (those with a decimal) are included within groups

Supplementary Table 4: PAM-derived sub-groups of *enn* genes

Enn sub-group	Enn alleles
SG1	enn74.0, enn74.1, enn74.2, enn79.0, enn79.1, enn79.2, enn80, enn81, enn82, enn83, enn84, enn85, enn86, enn87, enn88.0, enn88.1, enn90, enn91.0, enn91.1, enn91.2, enn92.0, enn92.1, enn94, enn95.0, enn95.1, enn97, enn98, enn99, enn100, enn101, enn102.0, enn102.1, enn102.2, enn105.0, enn105.1, enn107, enn108, enn110.0, enn110.1, enn110.2, enn113.0, enn113.1, enn114, enn115, enn116.0, enn116.1, enn118, enn119, enn120, enn121, enn122, enn123, enn124, enn125, enn126, enn127.0, enn127.1, enn129.0, enn129.1, enn129.2
SG2	enn132.0, enn132.1, enn132.2, enn132.3, enn136, enn137, enn138, enn139, enn140, enn141.0, enn141.1, enn141.2, enn144, enn145, enn146, enn148.0, enn148.1, enn148.2, enn150, enn152, enn153, enn154, enn155.0, enn155.1, enn157
SG3	enn2.0, enn2.1, enn3, enn5.0, enn5.1, enn5.2, enn5.3, enn5.4, enn10, enn11, enn13, enn14.0, enn14.1, enn16, enn20, enn22, enn23, enn26.0, enn26.1, enn28, enn29, enn30, enn31, enn32, enn33.0, enn33.1, enn33.2, enn34, enn35, enn36, enn37.0, enn37.1, enn38, enn40.0, enn40.1, enn40.2, enn40.3, enn40.4, enn44, enn45, enn48.0, enn48.1, enn51, enn52.0, enn52.1, enn52.2, enn52.3, enn55, enn56, enn57, enn58.0, enn58.1, enn60, enn61, enn62, enn68, enn354
SG4	enn12, enn17.0, enn17.1, enn19, enn21, enn47, enn63, enn64.0, enn64.1, enn66, enn67, enn69, enn70, enn71, enn356
SG5	enn230, enn231.0, enn231.1, enn232.0, enn232.1, enn234, enn235, enn237.0, enn237.1, enn237.2, enn240, enn241, enn242, enn243, enn244, enn245, enn246, enn247, enn248, enn249, enn250, enn251, enn162, enn163, enn164, enn165, enn166, enn167, enn168.0, enn168.1, enn170, enn171, enn172, enn173, enn174, enn175, enn176, enn177, enn179.0, enn179.1, enn181, enn212.0, enn212.1, enn213, enn214, enn215, enn216.0, enn216.1, enn218, enn219, enn333, enn344.0, enn344.1, enn345.0, enn345.1, enn346
SG6	enn178, enn182, enn183.0, enn183.1, enn183.2, enn183.3, enn187.0, enn187.1, enn189, enn190, enn191, enn192, enn193, enn194.0, enn194.1, enn196, enn197, enn198, enn199.0, enn199.1, enn201, enn202, enn203.0, enn203.1, enn205, enn206, enn207, enn208, enn209, enn210
SG7	enn252.0, enn252.1, enn252.2, enn254, enn255, enn256, enn257, enn259, enn220, enn221, enn222, enn260, enn261, enn262, enn263, enn264, enn265, enn266, enn267, enn268, enn269, enn271, enn272, enn274, enn275, enn276, enn277, enn278, enn308, enn316, enn317, enn347, enn348
SG8	enn223, enn224, enn225, enn226, enn227, enn291, enn292, enn294, enn295.0, enn295.1, enn297, enn318, enn319, enn320, enn321, enn322, enn323, enn324, enn325.0, enn325.1, enn327.0, enn327.1, enn336.0, enn336.1, enn336.2, enn337.0, enn337.1, enn339, enn340, enn341, enn342
SG9	enn284.0, enn284.1, enn286, enn287, enn288, enn289, enn290, enn270, enn273, enn279, enn299, enn300.0, enn300.1, enn302, enn303, enn304, enn305.0, enn305.1, enn307, enn309, enn310, enn311, enn312, enn313, enn314, enn315, enn355, enn293, enn298, enn329, enn330, enn331.0, enn331.1

Table 4: enn sub-group allele lists. Protein sequences were used to define sub-groups, but alleles which have the same mature protein sequences (those with a decimal) are included within groups

Supplementary Figure 1: k-means based clustering algorithm employed to determine the optimal number of sub-groups for M proteins. Although 4 sub-groups was the first number with the highest local maximum Gap score, 9 clusters had higher statistical support without excessive risk of spurious clustering.

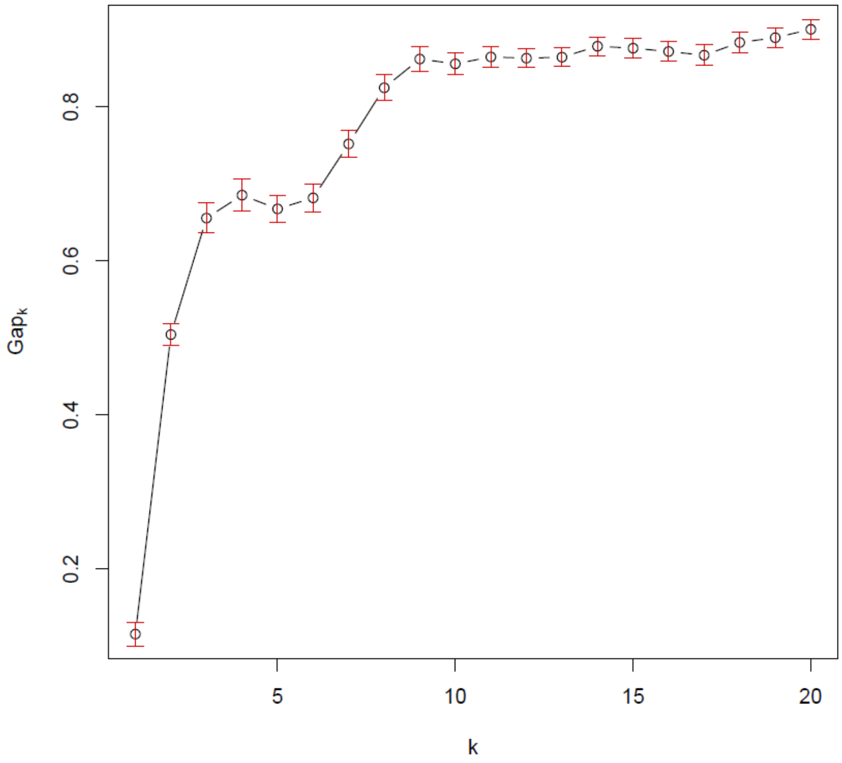

Supplementary Figure 2: Splitstree network and PAM sub-grouping of M proteins with previously published *emm* clusters overlaid. Sub-groups are represented by coloured ellipses, and the coloured node tips relate to *emm* cluster designations. Groups 1 and 2 correlate to clades X and Y. Cluster E6 is divided across sub-groups 4 and 5, and genetically remains between group 1 and the rest of group 2. Cluster D4 is also split into 2 subgroups (6 and 7) and has less overlap with the remaining M proteins from clade Y/group 1.

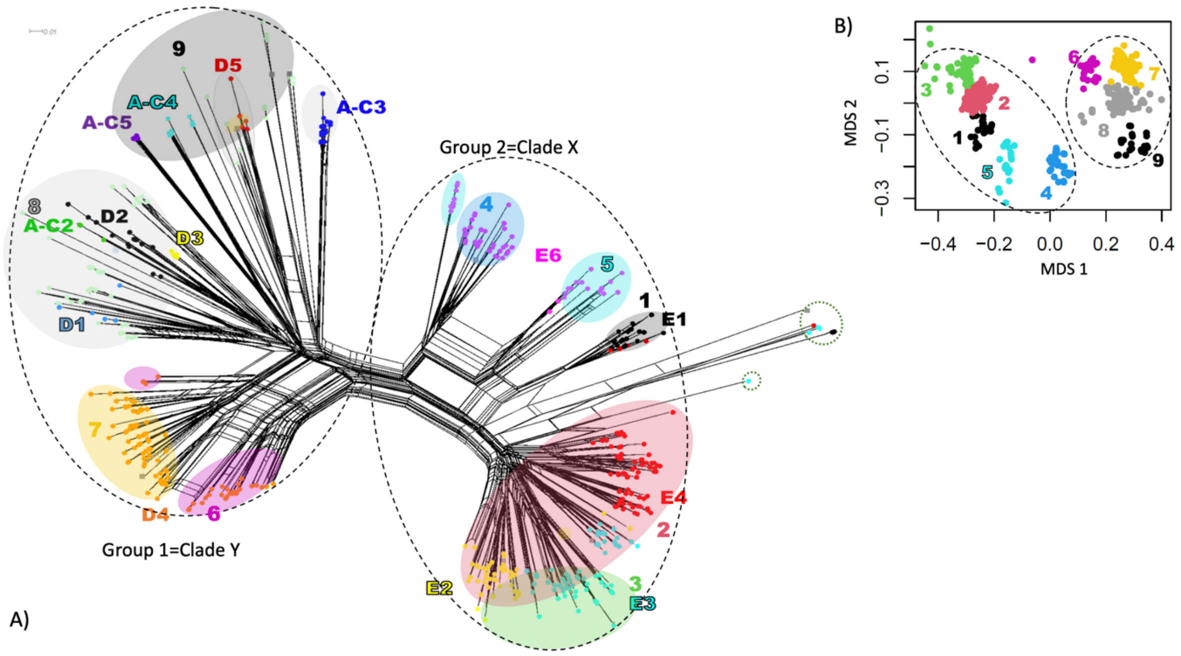

Supplementary Figure 3: A) Co-occurrence networks of *mrp* and *enn* alleles generated with igraph. Communities were detected with label propagation and had high modularity (0.9809). These networks and communities show that there are a higher number of discrete associations between a *mrp* and *enn* alleles (n=198 communities) than with *emm* type. Communities are distinguished by both node and surrounding cloud colour. Network analysis of Mrp (B) and Enn (C) sub-groups with the *emm*-cluster of the genome from which they originate. Nodes are sized proportionate to the number of genomes with each cluster or sub-group, and the thickness of connections is proportionate to the number of genomes with each combination. Community detection revealed 3 Mrp sub-group and emm-cluster groups (modularity = 0.41) and 5 Enn sub-group and emm-cluster groups (modularity = 0.43). Groups are distinguished by both node and surrounding cloud colour.

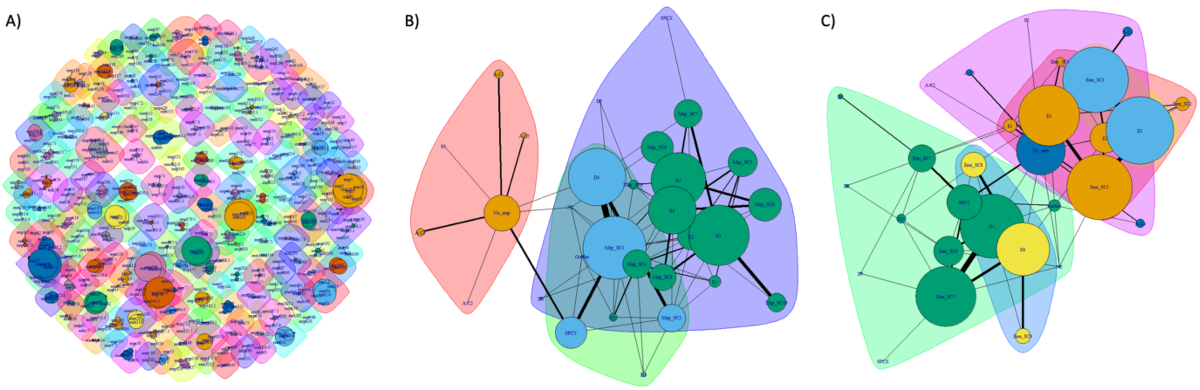

Supplement: Supplementary material 1 [file mic-169-1280-s001.pdf]
